# Violence Prevention Climate in General Adult Inpatient Mental Health Units: Validation study of the VPC‐14

**DOI:** 10.1111/inm.12750

**Published:** 2020-06-13

**Authors:** Geoffrey L. Dickens, Tracy Tabvuma, Kylie Hadfield, Nutmeg Hallett

**Affiliations:** ^1^ Centre for Applied Nursing Research Ingham Institute for Applied Medical Research Liverpool NSW Australia; ^2^ School of Nursing and Midwifery Western Sydney University Penrith South NSW Australia; ^3^ South Western Sydney Local Health District Liverpool NSW Australia; ^4^ School of Nursing University of Birmingham Birmingham UK

**Keywords:** aggression, psychometric tools, reliability, social climate, validity, violence, violence prevention

## Abstract

Ward social climate is an important contributor to patient outcomes in inpatient mental health services. Best understood as the general ‘vibe’ or ‘atmosphere’ on the unit, social climate has been subject to a significant research aimed at its quantification. One aspect of social climate, the violence prevention climate, describes the extent to which the ward is perceived as safe and protective against the occurrence of aggression by both the patients and the staff. The violence prevention climate scale (VPC‐14), developed in a UK forensic setting, was used in this study in a test of its validity in an Australian general mental health setting. The VPC‐14 was administered across eleven wards of one metropolitan Local Health District in Sydney, NSW. *N* = 213 valid responses from nursing staff and patients were returned (response rates 23.4 and 24.3%, respectively). The VPC‐14 demonstrated good internal reliability, and convergent validity was evidenced through moderate correlations with the WAS's anger and aggression subscale and the GMI total score. Concurrent validity was demonstrated by expected staff–patient differences in VPC‐14 rating and by correlations between incidents of conflict and containment on wards and the VPC‐14 ratings of staff and patients from those wards. Rasch analysis suggested that future tool development should focus on identifying ways to discriminate between ratings at the high end of the scale. The VPC‐14 supplies valid and useful information about the violence prevention climate in general adult mental health wards.

## Introduction

Violence is common in inpatient mental health settings where around 17.0% of inpatients commit at least one act during admission (Iozzino *et al.,* 2015). In turn, the restrictive practices that staff sometimes use to prevent violence including restraint and seclusion cause physical and psychological harm to all concerned (Bonner *et al.,* 2002; Fish & Hatton, 2017; Renwick *et al.,* 2016). Managing violence is costly, involving increased staffing levels, compensation claims, and related work absence (Bowers *et al*. 2011; Dickens *et al*. 2013). Prevention is, therefore, a vital part of the role of healthcare staff. Internationally, interpersonal violence has been recognized as a public health issue rather than one primarily for the criminal justice system (e.g. McDonald 2017). From this perspective, violence prevention comprises primary prevention activities taken before violence has occurred at policy, organizational, ward, and individual level; secondary preventative actions target avoidance of imminent violence (e.g. de‐escalation); while tertiary prevention encompasses interventions that are enacted while violence is occurring and in its aftermath to minimize harm (International Labor Office *et al*. 2002). Primary prevention is encapsulated in targeted and planned actions that are aimed at ameliorating the causal risk factors of violence. Therefore, identification and measurement of all relevant constructs plays a significant role in the determination of any links between antecedents and subsequent violence, and in investigations of interventions aimed at targeting risk factors to ameliorate their negative consequences.

## Background

One contributor to the complex interplay of factors preceding inpatient aggression is the ‘ward climate’, sometimes termed the ‘ward atmosphere’ or ‘ward environment’. In 1953, the World Health Organisation (1953 p.17) stated that ‘the single most important factor in the efficacy of treatment given in a mental hospital appears… to be an intangible element which can only be described as its atmosphere’. Importantly, ward climate has been found to be associated with numerous relevant outcomes in healthcare settings including staff burnout (Bowers *et al.,* 2009), stress (Redfearn *et al.,* 2002), job satisfaction (Tuvesson *et al.,* 2011), levels of disturbed patient behaviour (Bouras *et al.,* 1982), and perceived violence risk (Dickens *et al.,* 2014). In mental health inpatient settings, the Ward Atmosphere Scale (WAS; Moos, 1974) is the most widely used measure of climate. However, it lacks robust psychometric properties (Røssberg & Friis, 2003) and is lengthy (100 items in its most used form) and evidence about its psychometric properties is 'mixed' (Tonkin, 2015). Resulting from these perceived shortcomings, Schalast *et al*. (2008) developed the Essen Climate Evaluation Scale (EssenCES) to measure the ward atmosphere specifically in forensic mental health settings. While it has been used in non‐forensic settings (e.g. Baumgardt *et al.,* 2019), it has received no further validation for this purpose.

Reviewing the international literature, Hallett *et al*. (2014) identified the lack of an environmental climate measure specifically aimed at tapping into the unique ‘violence prevention climate’ of a ward: that is the things that get done by the staff and the patients on a ward that prevent violence (Spector *et al.,* 2007). Subsequently, they produced a valid and reliable psychometric tool (VPC‐14; Hallett *et al.,* 2018) to measure the phenomenon. Since its initial validation was conducted in a UK forensic setting, the current study aimed to test the VPC‐14 in the Australian acute public mental health inpatient sector to ensure its applicability. Specific objectives included the following: testing scale assumptions, targeting, internal consistency, convergent and concurrent validity, and sensitivity and stability. A further aim was to make any necessary empirically informed recommendations for future development of the VPC‐14 for use in adult general mental health settings.

## Methods

### Design

Techniques derived from classical test theory and item response theory were used to address the study objectives. Data were gathered in cross‐sectional and longitudinal surveys, partly conducted during an implementation of the Safewards conflict and containment reduction programme (Bowers *et al.,* 2014). The implementation and evaluation was informed by a steering group comprising consumer, academic, clinical, and executive nursing representation. Sample size was essentially pragmatic given the single local health district setting and execution of the study in the context of Safewards introduction.

### Setting and participants

The study was conducted across all eleven inpatient mental health wards of three hospitals in a culturally and linguistically diverse metropolitan local health district (population served: 1 million) in New South Wales. Eligible participants were all nursing staff and current inpatients deemed by nursing staff as able to provide implied consent by completing and returning anonymous survey questionnaires.

### Measures

#### VPC‐14 (Hallett et al., 2018)

This is a 14‐item scale developed based on extensive systematic and conceptual literature reviews of the perceptions of staff and patients regarding violence prevention in mental health settings (Hallett & Dickens, 2017; Hallett *et al.,* 2014). In its UK validation study, it demonstrated good internal reliability, convergent validity, and test–retest reliability (Hallett *et al*. (2018). Analysis revealed a two‐factor structure comprising ‘patient actions’ (things patients do that prevent violence) and ‘staff actions’ (the things that staff do). Before the current study, the tool was reviewed by the project steering group and numerous suggestions for item amendment were made. However, the group agreed that changes at this stage should be minimal and just one alteration was made. Item 3 ('Staff on the ward are good at talking down aggressive patients') was viewed as lacking in clarity; specifically, the term 'talking down' (intended to mean ‘de‐escalate’) was taken to mean 'talking down to' (i.e. 'in a condescending manner'). Therefore, we added a footnote to explain that 'talking down' was intended to mean 'in a way that intends to calm someone down or de‐escalate them'. Items are rated on a 5‐point Likert scale (Strongly Agree to Strongly Disagree).

#### Ward atmosphere scale (WAS; Moos, 1974) anger and aggression subscale

For the current study, we used the WAS' 10‐item Anger and Aggression subscale (typical item: ‘Patients here rarely become angry’). In the original scale, items are rated dichotomously (Yes/No) but the tool has previously been adapted to be scored on a 4‐point scale (Totally disagree to Totally agree) to facilitate expression of strength of agreement. This approach was used here.

#### Good milieu index (GMI; Friis, 1986)

This is a 5‐item index designed to quantify how positively respondents (patients/staff) rate a milieu (typical item: ‘In general how satisfied are you with the ward'). Total scores have been shown to be significantly correlated in expected directions with eight of ten WAS subscales (Friis, 1986). Items are rated on a 4‐point scale. Internal reliability was good (α = 0.845) in the current study.

#### Patient‐staff conflict checklist shift report (PCC‐SR; Bowers et al., 2005*)*

Numbers of incidents of conflict (e.g. aggression, rule‐breaking) and containment (e.g. seclusion/restraint) were extracted from shift reports on the PCC‐SR, a tool developed by Bowers et al. (2005) and used routinely on the wards under study during the current investigation. Internal reliability for conflict items was acceptable (Cronbach’s α = .730). Internal reliability across different types of containment incident would not be expected since each is known to have its own unique profile in terms of overall acceptability for management of both aggressive and self‐harming behaviours (Bowers et al., 2007; Hosie & Dickens, 2018).

#### Demographics

Respondents were asked to provide their staff/ patient status, and ward.

### Procedure

The study was approved by the South Western Sydney Local Health District Human Research Ethics Committee (Ref: 2019/ETH10615). Data were collected between April 2019 and January 2020 during the implementation of the Safewards conflict and containment reduction programme (Bowers *et al.,* 2015) in eight of eleven mental health wards. However, patients and staff on non‐participating wards were offered the opportunity to participate in the VPC‐14 validation study. On participating wards VPC‐14, WAS Anger and Aggression subscale, and GMI data collection, was conducted 4 weeks prior to Safewards commencement; the VPC‐14 was readministered 4 weeks following completion of the implementation of the Safewards implementation (i.e. 24 weeks later). On wards not participating in Safewards, data collection (VPC‐14, WAS, and GMI) was conducted once only. For questionnaire data collection, potential participants were informed of the study and its purpose through posters and participant information sheets. For staff, the nurse manager of each unit was provided with sufficient information sheets and study questionnaires for circulation. For patients, one of the study team (TT) attended wards to offer assistance if requested. PCC‐SR data were gathered for the 4‐week period preceding the first (or only) iteration of questionnaires on each ward and, on Safewards implementing wards, for the 4‐week period following implementation.

### Analysis

Data were entered into IBM SPSS Statistics Version 25.00.1 (IBMCorp, 1989, 2017). Because survey questionnaires were distributed by Nurse Unit Managers and not addressed to individual‐named respondents, we did not have a precise count of all those who were provided with study materials. We, therefore, calculated response rate as the number of valid returned questionnaires divided by the number of nurses employed and patients resident on wards at the time of survey distribution. Analytic strategies were as below:

#### Scale assumptions

Responses were described in terms of score distribution, floor, and ceiling effects.

#### Internal reliability

Cronbach’s alpha (α) was calculated for all scale/subscale scores. The following rubric was used to determine levels of internal consistency: 0.7 acceptable, 0.8–0.89, good (George & Mallery, 2003).

#### Convergent validity

Pearson’s correlations (*r*) were calculated between the VPC‐14 total score and its constituent ‘staff actions’ and ‘patient actions’ subscales, the WAS Anger and Aggression subscale (Moos, 1974) and the GMI (Friis, 1986).

#### Concurrent validity

Previous research has reported significantly different ratings between staff and patients on climate measures (Baumgardt *et al.,* 2019; Berry *et al.,* 2016; Moos, 1974; Nicholls *et al.,* 2015; Southard *et al* (2012); ; ; ; ; ; ; ; . While the literature is not without inconsistencies, the weight of evidence across settings and measures is that, relative to patients, staff overestimate their own abilities as active agents for therapeutic change and that patients, relative to staff, perceive a greater role for themselves in determining ward climate. This led us to hypothesize that, for the VPC‐14, differences between staff and patients' ratings would be revealed at the level of ‘staff actions’ and ‘patient actions’ factors: hypothesis i: staff will score higher than patients on the ‘staff actions’ VPC‐14 factor; hypothesis ii: patients will score higher than staff on the ‘patient actions’ factor. These hypotheses were tested using independent samples *t*‐tests. A second test of concurrent validity draws on the assumption that one would expect the quality of the violence prevention climate to reflect the actual level of conflict and containment on the ward. We, therefore, anticipated negative correlations between VPC‐14 scores of individuals and the overall occurrence of both conflict and containment on their ward (hypothesis iii: a better perceived violence prevention climate on a ward should be associated with lower conflict and lower containment). To account for different response rates and bed numbers between wards, the data about conflict and containment for these analyses were standardized by calculating the mean number of incidents per returned PCC‐SR form divided by the number of beds on the ward. To test this hypothesis, correlation analysis (Spearman’s ρ) was conducted to examine the relationship between scores of individuals on the VPC‐14 and the adjusted ward level conflict and containment rates.

#### Sensitivity/stability

Test–retest reliability for the VPC‐14 has been previously addressed (Hallett *et al.,* 2018) and was not replicated in the current study given the complexities of testing respondents at an interval while maintaining their anonymity. However, we would expect that improvements in a likely external determinant of ward climate would be reflected in ward climate ratings. We, therefore, calculated conflict and containment rates (mean incidents per completed form/ number of beds on ward) for the four weeks following the Safewards implementation period on seven of the eight participating wards. Due to an oversight, the eighth participating ward was not provided with pre‐implementation VPC‐14 surveys. These were compared with equivalent data for the four weeks prior to implementation (see concurrent validity above) to determine whether an actual change had occurred which we might expect to be reflected in WAS scores. This resulted in identification of a 29.1% reduction in reported conflict incidents and a 14.6% drop in containment incidents per PCC‐SR/bed day across the relevant wards. To test whether this had been reflected in VPC‐14 ratings, the scores of respondents on the relevant wards were compared at iteration one and iteration two using independent samples*t*‐tests.

#### Diagnostic testing (Rasch analysis)

**Fig. 1:**
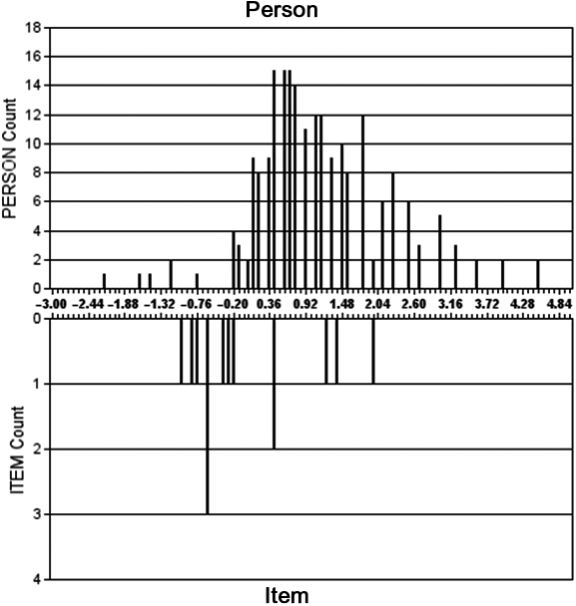
Figure 1 shows distribution of person ability (top) mapped against distribution of item ability (below) along a scale representing score in logits (SDs). Clustering of 6 items between −1.32 and −0.20 logits indicates possible redundancy since all these items only uniquely capture the score of one individual. An absence of items targeting individuals who score above 2.0 logits suggests that the tool may lack sufficiently ‘difficult’ items to adequately distinguish those who score higher than this.

Rasch methods are used to determine whether a scale measures the full range of the construct under examination. This can be visualized as a ruler (see Fig. 1) measuring scale items in terms of their difficulty (from left to right less to more) and respondents in terms of their ‘ability’ (in tests of agreement like the VPC‐14 ability should be taken to mean the extent to which respondents find it difficult to agree with each item). Rasch analysis informs about the following issues:

- Fit: describes the extent to which item rating is congruent with the imputed meaning of item scores (i.e. are mean item‐ability scores monotonically ordered) and whether items work together to capture each individual's level of ‘performance’. Key indicators of good fit are the MNSQ infit and outfit statistics with values of 0.6‐1.4 representing the ideal range with scores as low or high as 0.5 or 2.0 potentially degrading the validity of the scale (Linacre, 2012). Low person separation/reliability (<2, <0.8) or low item separation/reliability (<3, <0.9) imply inability of a tool to discriminate between low and high scorers and insufficient sample size, respectively.
- Targeting: describes how people and items are distributed along the perceived violence prevention climate continuum and whether the VPC‐14’s constituent items cover the full range of the person distribution. Examination informs whether items are redundant due to multiple items covering the same part of the difficulty distribution.
- Dimensionality: Principal components analysis of the residuals (PCAr) is used to assess unidimensionality or suggests whether underlying latent traits should be investigated.
- Item invariance: Differential item functioning (DIF) identifies whether different groups of individuals (e.g. nursing staff and patients) respond in any systematically different way.

## Results

*N* = 228 VPC‐14 questionnaires were returned. Following identification of missing data, *N* = 213 responses were deemed admissible, 78 from patients and 135 from nursing staff (response rate 25.4% and 25.3%, respectively). *N* = 123 responses came from acute wards (*n* = 81 staff, *n* = 42 patients) and *N* = 90 from non‐acute wards (*n* = 54 patients, *n* = 36 staff). *N* = 92 responses came from seven of the eight wards on which Safewards was to be implemented on iteration one; a further *n* = 90 came from the same wards on iteration two 24 weeks later. The remaining *n* = 31 responses came from three wards that did not participate in Safewards, and one ward where the pre‐Safewards VPC‐14 iteration was not conducted. Table 1 summarizes results relating to the psychometric properties of the VPC‐14.

**Table 1:** Summary of results of psychometric evaluation of VPC‐14

Psychometric property	Total (%)	
Missing data (%)		
Initial	56/3206 (1.75%)	
Respondents with missing data	30/228 (13.2%)	
Respondents with> 10% missing data (Total missing items 39) and excluded	15/228 (6.6%, *Mdn = *2, range = 2 – 14)	
Respondents with 1 missing item only [and missing data imputed]	15/213 (0.6% of 2982 data points)	
Scale assumptions		
Item scores: *M* (*SD*, range) [Item]	3.75 (0.89, 2.48 [7] to 4.27 [8])	
Item SD range [Item]	0.72 [8]–1.09 [7]	
Targeting		
*M* Total Score (*SD*)	52.52 (7.12)	
Possible score range	12–72	
Observed score range	23–68	
Floor/ Ceiling effect	0/0	
Rating scale score (of 2982)^†^	1	123 (4.1%)
	2	274 (9.19%)
	3	552 (18.51%)
	4	1306 (43.8%)
	5	727 (24.38%)
Reliability		
Cronbach's α (whole scale)	0.836	
Improvement if [item] removed	0.839 [5] 0.845 [7] 0.837 [9]	
‘Staff actions’ factor [1,2,4,6,8,10,12,13, 14]	0.888	
Improvement if [item] removed	0.899 [13]	
‘Patient actions’ factor [3,5,7,9,11]	0.689	
Improvement if [item] removed	0.697 [7] then 0.709 [9] (Whole scale α = 0.854 with 7,9 removed; α = 0.857 with 13 removed also)	
Mean item–item correlation		
Convergent validity		
Correlation with WAS (N = 106)	*r* = 0.448 (*P *< 0.001), *r = 0*.211 (*P *< 0.05), *r *= 0.566 *P *< 0.001	
Correlation with GMI (N = 112)	*r* = 0.459 (*P *< 0.001), *r *= 0.472, *P *< 0.001, *r *= 0.235, *P < *05	
VPC Total, ‘Staff actions’, ‘Patient actions’		
*Sensitivity/ Stability*		
Iteration 1 (n = 92) vs. Iteration 2 (n = 90) on		
Participating Safewards units		
VPC Total	52.62 (5.92) v 52.48 (8.19) *t* = 0.133, d.f. = 180 *P *= 0.894	
‘Staff actions’	37.00 (4.30) v 37.01 (6.24) *t* = −0.22, d.f. = 180 *P *= 0.983	
‘Patient actions’	15.62 (3.19) v 15.47 (3.67) *t* = 0.309, d.f. = 180 *P *= 0.758	
Concurrent validity		
H1 Staff (n = 135) v Patient (n = 78)		
‘Staff actions’	37.05 (5.28) v 36.81 (5.49) *t* = 0.319, d.f. = 319, *P *= 0.75	
‘Patient actions’	14.64 (3.49) v 17.14 (2.73) *t* = −5.434, d.f. = 211 *P *< 0.001 *d* = 0.80	
H2 Acute (*n* = 123) v Non acute wards (*n* = 90)		
‘Staff actions’	36.51 (5.55) v 37.58 (5.02) *t* = 1.457, d.f. = 211 *P* = 0.147	
‘Patient actions’	14.73 (3.40) v 16.69 (3.20) *t* = 4.255, d.f. = 211, *P *< 0.001	
VPC Total	51.24 (7.15) v 54.27 (6.73) *t* = 0.713, d.f. = 211, *P *= 0.002	
H3 Relationship between VPC‐14 ratings and recorded conflict and containment		
‘Staff actions’ with recorded conflict	ρ = −0.273, *P *< 0.01	
‘Patient actions’ with recorded conflict	ρ = −0.186, *P *< 0.05	

^†^ Items 7, 9, and 13 are worded such that agreement denotes poor violence prevention climate and are reverse‐scored; '5' is always the most desirable and '1' the least desirable rating.

### VPC‐14 Data quality

On all completed questionnaires, there were 1.75% (56/3206 from 30 respondents, range 1–14) missing items. Those (*n* = 15) who had missed >1 (i.e. >10.0%) items were excluded casewise leaving a total of *N* = 213 completed VPCs of which *n* = 15 had one missing data item (0.6%). Those with missing items were not disproportionately represented on their staff/patient status, wave 1 or 2 data collection, or acute non‐acute clinical area status. No VPC‐14 item was over‐represented among missing values (10 [range 0‐3] of the VPC‐14 items were among the missing values). Thus, 17 items were assumed to be missing completely at random and were replaced through multiple imputation.

### Data distribution

The VPC‐14 total score was moderately negatively skewed (Skewness = −0.575) and somewhat leptokurtic (1.952). The ‘staff actions’ factor was moderately skewed (−1.381) and also leptokurtic (4.966). The ‘patient actions’ factor was within the acceptable range for skewness (−0.250) and kurtosis (0.448).

### Internal reliability

Whole scale reliability was very good (α = 0.836) with only marginal improvements possible through item deletion. ‘Staff actions’ factor reliability (α = 0.888) was very good and only marginally improvable through item deletion (Item 13). The ‘patient actions’ factor was marginally below the acceptable threshold (α = 0.689) but was improved by deletion of items 7 and 9 (α = 0.709) which also improved whole scale reliability (α = 0.854 for the resulting 12‐item scale).

### Concurrent validity

#### Staff vs. patient ratings

Table 1 shows that staff and patients did not differ significantly in terms of their ratings of the ‘staff actions’ factor of the VPC‐14. Conversely, patients rated ‘patient actions’ significantly more favourably on their ward than did staff.

#### VPC‐14 rating relationship with conflict and containment data

The VPC‐14 ‘patient actions’ factor was significantly negatively correlated with incidence of both conflict (ρ = −0.273, *P* < 0.01) and containment (ρ = −0.186, *P* < 0.05) in the 4 weeks prior to VPC‐14 data collection. The ‘staff actions’ factor was not significantly associated with either conflict or containment.

#### Sensitivity to change

There was no difference in VPC‐14 total or subscale scores from iteration one and iteration two from respondents on wards which had participated in Safewards despite the reduction in conflict and containment across the period.

### Rasch analysis

#### Fit

Visual inspection of probability category curves and item threshold scores revealed that for item 13 (‘Staff are rude to patients’), the ordinal numbering of responses (2‐3‐1‐4‐5) was incongruent with the imputed meaning. MNSQ infit and outfit statistics, indicating the overall fit of items to the Rasch model, mostly fell within the acceptable range (0.6 to 1.4). Exceptions were Items 7 and 13 (infit 1.53 and 1.49, respectively) and 7, 13, and 5 (outfit 1.65, 1.55. and 1.54, respectively). They did not exceed 2.0 and were unlikely to degrade the measurement scale. Person separation (4.25) and reliability (0.95) and item separation (13.59) and reliability (0.99) indices were all within boundaries suggesting good internal reliability and sufficient sample size.

#### Targeting

Items successfully captured 174 (81.7%) of the sample (see Fig. 1). At the low end of the scale were five individuals who were not adequately targeted by the tool (i.e. items did not adequately capture how poorly they rated the climate; and any further decline in their perceptions would not be detected in an iterated measurement). Additionally, 6 items were required to target just one individual (i.e. removal of any or all six items would have little detriment to targeting at the low end of the scoring range]. At the high end, items failed to adequately target the performance of 34 individuals. In other words, the scale did not adequately capture how positively they viewed the ward violence prevention climate and any improvements in these individuals’ perceptions would be unlikely to be captured in an iterated measurement. To investigate whether deletion of apparently redundant items (i.e. those measuring same part of ‘ability’ spectrum), we calculated internal reliability for a scale without the ‘staff actions’ items 2, 6, 12, and 13. This resulted in Cronbach’s α = 0.833 for a 5‐item ‘staff actions scale’ and of α = 0.750 for a whole scale 10‐item VPC with the original five ‘patient actions’ items appended.

#### Dependency

Residual correlations approached but did not exceed 0.7 (range −0.06 to 0.670 [4/91 correlations >0.6]) indicating no local dependency between pairs of items or persons.

#### Dimensionality

**Fig. 2:**
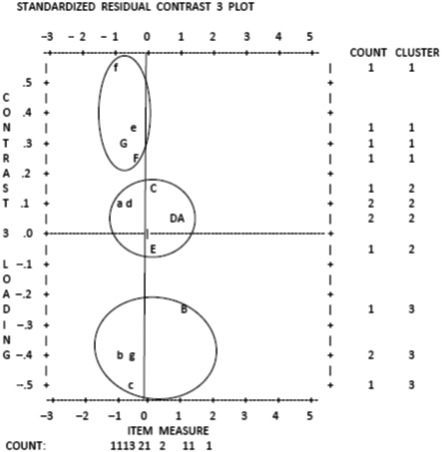
Cluster 1: ‘Practical staff availability’: G, Staff are good at listening to patients [VPC01]; f, Staff on this ward show the patients respect [VPC08]; e, There is usually a member of staff around for patients to talk to [VPC12]; F, Staff are rude to patients [VPC13]. Cluster 2: ‘Patient issues’: E, Patients on this ward show the staff respect [VPC03]; D, Patients are good at controlling their inner feelings [VPC05]; d, Staff here have a good knowledge of the patients [VPC06]; A, Patients bully other patients [VPC09]; a, Staff know when to intervene when a patient is becoming aggressive [VPC10]; C, Patients are nice to each other [VPC11]. Cluster 3: ‘Staff de‐escalation skills’: b, The staff here are experienced in preventing aggression [VPC02]; c, Staff on the ward are good at talking down aggressive patients [VPC04]; B, Patients sometimes annoy other patients on purpose [VPC07]; g, Negotiation with aggressive patients is used effectively by staff [VPC14].

Principal components analysis of the residuals (PCAr) not is not like a usual factor analysis and does not show loadings on one factor, rather it highlights contrasts between opposing factors and can suggest that a secondary dimension may be at play. Analysis revealed an Eigen value of 3.4268 at the first contrast indicating that the VPC‐14 was multidimensional and suggesting that the pattern of rating of three items contrasts strongly with that of the remaining items. Visual inspection of the standardized residual contrast plot suggested that the outlying items were 2, 4, and 7 and suggested three‐item clusters (see Fig. 2). Eigen values for each cluster at the first contrast were ≤2 for factors 1 and 2 (1.69, 1.84, and 2.00, respectively) confirming the unidimensionality of each. The raw variance explained by each factor at the first contrast was, in order, 44.9%, 52.8%, and 62.0% and should, therefore, be considered strong measurement dimensions (Conrad *et al.,* 2011). Further investigation revealed internal reliability for each of the three clusters, α= .735 (.770 if item 13 removed), α = 0.684 (0.709 if items 6, 9, and 10 deleted) and α = 0.609 (.804 if item 14 deleted). Deletion of all reliability‐maximizing items results in a 9‐item scale (α = 0.757). Clusters were agreed to relate to staff's practical availability to patients, patient issues, and staff de‐escalation skills.

#### Item invariance

Comparison of responses by staff and patients and by acute versus non acute ward revealed no significant item invariance with all contrasts falling below the 0.64 logit criterion (Staff vs. patients DIF Contrast range ‐.48 ‐ .61; acute versus non‐acute DIF contrast range −0.62 to 0.58) suggesting the same tool is fit for purpose across groups and settings.

## Discussion

We aimed to determine the validity of the VPC‐14 for use in Australian general, adult mental health inpatient settings. Despite consensus that ward climate is an important determinant of patient outcomes, there is a lack of agreement on what is the best measure of social climate in the general as opposed to forensic inpatient settings. The commonly used WAS is lengthy (100‐items) and has limited evidence regarding its underlying factor structure. A more recent tool, the EssenCES (Schalast *et al.,* 2008), developed in Germany and the UK for use in forensic settings, has been used in non‐forensic settings (e.g. Kerfoot *et al.,* 2012 in the UK, Baumgardt *et al.,* 2019 in Germany) but has not been validated for such use. In the context of the implementation of the Safewards conflict and containment programme in one Australian Local Health District, and the need to evaluate the success or otherwise of the intervention impacting on social climate as well as on actual conflict and containment rates, we investigated the properties of the VPC‐14.

The internal reliability of the whole scale and ‘staff actions’ factor were very good (α = 0.836); ‘patient actions’ fell short of the acceptable threshold, but this was reversed by removal of items related to deliberate provocation (item 7) and bullying (item 9). Manifestations of these two items may well differ in forensic settings like that of the VPC‐14 validation study. Nevertheless, convergent validity for the underlying VPC‐14 constructs was evidenced through moderate correlations with the WAS Anger and Aggression subscale and the GMI, a measure with correlations with all aspects of the WAS. Additionally, predictions about likely differences between staff and patient ratings on ‘staff actions’ and ‘patient actions’ were supported for the latter with ‘patient actions’ being rated more highly by patients than staff. However, ‘staff actions’ were not rated differently by the two groups. This is worth further exploration, and it should be noted that previous research in this area is not unequivocal. Results do not fly in the face of accepted truth, but rather simply fail to support a logical hypothesis which has some empirical support (e.g. Berry *et al,* 2016). Concurrent validity for the VPC‐14 scales was suggested through demonstration of a significant association with actual recorded conflict and containment in the expected directions, that is better climate on less conflict and containment‐heavy wards.

Regarding tool sensitivity, a real world change in conflict and containment, that is reduction in incidents of conflict and containment, was not reflected in the VPC‐14 total or subscale scores from repeat iterations on Safewards implementing units. This was disappointing, though there are a number of potential explanations. We have taken significant reductions in conflict and containment to indicate that a social climate measure ‘should’ reflect that; however, the analysis takes no account of differing perceptions of subgroups of respondents, nor of the variations in reductions of conflict and containment across wards. Further, violence prevention climate may require a longer period of sustained improvement in actual conflict and containment rates before it reflects change. Much of the research into the effect of intervention on ward climate change in adult mental health settings has failed to detect significant differences in response to targeted interventions. Finally, the climate measurement tool may actually be insensitive to actual change. These issues are discussed in light of the Rasch analysis in the next section. To conclude this section, traditional testing of the VPC‐14 suggested that it is a valid and reliable tool for measuring the violence prevention climate in the current study setting.

Given its development in a forensic setting, it is perhaps unsurprising that a number of issues have arisen when using the VPC‐14 to measure ward climate in a general mental health setting; additionally, cultural differences between UK and Australian settings may have contributed. Notable issues diagnosed by Rasch analysis include the incongruent scoring of item 13 (‘Staff are rude to patients’). This may suffer from being a reverse‐scored item for which respondents have to change ‘mind set’ in relation to the scoring order. Interestingly, the other reverse‐scored items (7 and 9) were detrimental to the internal reliability of the ‘patient actions’ subscales which may support this view. Simple deletion of item 13 seemed to have little adverse effect on the scale, and one solution could simply be to omit it. An additional issue highlighted by Rasch analysis was the clustering of scale items in terms of their ability to discriminate between respondents on ‘ability’ (i.e. their individual overall rating of the ward climate). Ideally, a scale will comprise items of varying ‘difficulty’ such that, when ordered, it is increasingly hard for an individual to rate successive items as highly as the last. In this way, the scale will cover the maximum range of ability among respondents and will maximize the level of discrimination that can be made between respondents based on their responses. The clustering evident in the current Rasch model suggested that a number of items were redundant; indeed, deleting them from the scale had no noticeable adverse effect on internal reliability. Simultaneously, the highest scoring 20% of individuals were inadequately captured by scale items. Efforts to measure any improvement in social climate for these individuals could, therefore, run the risk of failing to capture that change because they have limited room for their scores to improve. While ceiling effects for the tool were not obvious from descriptive statistics (i.e. no individuals scored a maximum 70 points), the moderate skewness and leptokurtic distribution does seem to play a part in this targeting issue. A two‐pronged solution in terms of future development of the VPC‐14 is suggested. In order to increase the targeting range, new items may need to be generated which are more 'difficult' for individuals to rate with the greatest positivity This may be aided by lengthening of the response scale, for example to a seven‐point measure as per the EssenCES (Schalast *et al.,* 2008). However, evidence for any great benefits from such amendment is mixed (Krosnick & Presser, 2010).

The study has some limitations. We treated repeated iterations of VPC‐14 which comprise some of the data set as independent when some may have been completed by the same individuals twice. This was because we collected no identifying information about respondents and could theoretically inflate some indicators such as internal reliability. Response rates of 25.4% (staff) and 25.3% (patients) were far from ideal and were also a limitation; however, they may go some way to demonstrating that participation in both iterations was not very widespread. It was also probably more common in nursing staff due to patient turnover across the 20‐week period between iterations. Nursing staff frequently informed the research team that they were disinclined to participate because they were ‘too busy’ and they also appeared to exercise a significant gate‐keeping effect on patients' participation by informing the team that they were too disturbed to be approached. All inferential statistics involving VPC total, ‘staff actions’, and ‘patient actions’ scores have been derived from the original 14‐, 9‐, and 5‐ item version only and not from newly derived scores based on current analyses which are, essentially, suggestions for amendments informed by the diagnostic value of Rasch analysis. It should be reiterated that the extensive psychometric properties of the tool do refer to its current version and that any future amendments will need to ensure these properties are not degraded.

## Conclusion

The VPC‐14 supplies valid and useful information about the violence prevention climate in general adult mental health wards. We recommend further exploration and development to maximize its ability to detect change over time. Conceptualization of the violence prevention climate as comprising three distinct areas, staff availability, patient issues, and perceived staff de‐escalation skills, may assist communication of the basic fact that much de‐escalation work occurs simply in the 'being there for' patients.

## Relevance for clinical practice

Maintenance of a therapeutic environment is a key role of mental health nurses. The VPC‐14 offers a valid, reliable, and simple way of monitoring the social climate which is a key element of that environment. Additionally, it offers an opportunity to measure the impact of initiatives and developments to improve the environment.

## Funding

The current study was funded by a Strategic Reserve Funding grant from the Nursing and Midwifery Office, New South Wales Government. The funder played no part in the design, conduct, data collection, analysis or in the decision to publish or the content of any publication.

## Ethics Approval Statement

The study was approved by the South Western Sydney Local Health District Human Research Ethics Committee (Ref: 2019/ETH10615).
